# Alterations of Gut Mycobiota Profiles in Adenoma and Colorectal Cancer

**DOI:** 10.3389/fcimb.2022.839435

**Published:** 2022-02-24

**Authors:** Renyuan Gao, Kai Xia, Minkang Wu, Hui Zhong, Jing Sun, Yin Zhu, Linsheng Huang, Xiaocai Wu, Lu Yin, Rong Yang, Chunqiu Chen, Huanlong Qin

**Affiliations:** ^1^Diagnostic and Treatment Center for Refractory Diseases of Abdomen Surgery, Department of General Surgery, Shanghai Tenth People’s Hospital, Tongji University School of Medicine, Shanghai, China; ^2^Department of Pediatrics, Shanghai Tenth People’s Hospital, Tongji University School of Medicine, Shanghai, China

**Keywords:** gut mycobiota, metagenomic sequencing, microbial network, diagnostic model, colorectal cancer

## Abstract

Accumulating evidence indicates that gut microbiota dysbiosis contributes to colorectal cancer and adenoma. However, a few studies revealed the altered gut mycobiota architecture in colorectal cancer. The present study characterized the gut mycobiota profiles in adenoma and colorectal cancer patients by metagenomic sequencing. *Malassezia restricta* increased, while *Leucoagaricus_sp_SymCcos* and *fungal_sp_ARF18* significantly decreased in adenoma. *Phanerochaete_chrysosporium*, *Lachancea_waltii*, and *Aspergillus_rambellii* were the top 3 fungi that were significantly enriched in colorectal cancer, while *Candida_versatilis*, *Pseudocercospora_pini_densiflorae*, and *Candida_sp_JCM_15000* were dominant in the healthy controls. Thirteen fungi, ranked as critical biomarkers in diagnosing colorectal cancer, showed positive associations among all samples. *Lachancea_waltii* and *Phanerochaete_chrysosporium* showed the most significant association within CRC. The values of area under the receiver-operating characteristics curve (AUROC) of selected 13 mycobiota were 0.926 in the training model and 0.757 in the 10-fold validation model. Our study provided a reliable investigation of the alterations of gut mycobiota in the development of colorectal cancer and established a convincing diagnostic model for colorectal cancer, which might improve the treatment strategy for colorectal cancer in the future.

## Introduction

Colorectal cancer is one of the most common digestive tract cancers worldwide that threatens the life of millions of human beings every year (Siegel et al., 2020). The increasing trend of morbidity and poor prognosis of early-onset colorectal cancer was another novel challenge observed by the latest epidemiology survey (Peters et al., 2015; Akimoto et al., 2020). Massive public health resources and attention have been paid to the prevention and early detection of colorectal cancer (CRC) to reduce the enormous consumption of medical resources and improve the clinical prognosis in developed and developing countries. However, many factors, such as obesity, high-fat diet, sedentary behavior, genetics, and diabetes, acted as evil backstage manipulators to promote the occurrence of CRC (Han et al., 2014; Simons et al., 2014; Zhang et al., 2015; Dabrowski et al., 2016; Keum and Giovannucci, 2019). Of note, the gut microbiota was considered as one of the most valuable and controllable carcinogens in the occurrence and development of CRC recently (Gao et al., 2017a; Kang and Martin, 2017).

Bacteria, which accounted for 99% of all the microorganisms, dominated and functioned in the gut to maintain the whole body’s health. Many clinical samples and exquisitely designed animal experiments provided solid evidence on the carcinogenic roles of certain enriched bacteria, such as *Fusobacterium nucleatum* and *pks+ Escherichia coli*, in CRC patients. The sophisticated mechanisms of the signal transduction pathways triggered by these harmful carcinogens in the gut were also revealed during the past few years. However, a limited number of studies focused on the roles of mycobiota in colorectal cancer.

Although the mycobiota only accounted for less than 0.1% of the number of total microorganisms in the gastrointestinal tract, they acted as essential immunoregulators for microenvironment homeostasis and remote organs (Rizzetto et al., 2014; Underhill and Iliev, 2014; Halwachs et al., 2017). Alterations of fungal composition were likely to cause immune dysfunction and mucosal barrier impairment, triggering an outbreak of systemic disorders (Iliev and Leonardi, 2017). For instance, the fungi *Candida*, such as *Candida tropicalis* and *Candida glabrata*, were abundant in inflammatory disease (Liguori et al., 2016; Richard and Sokol, 2019). *Malassezia sympodialis* and *Saccharomyces cerevisiae* were also enriched in inflammatory bowel disease (Sokol et al., 2016). Fungal dysbiosis was also prevalent during carcinogenesis. We previously observed an increased proportion of *Trichosporon* spp. and *Malassezia* spp. in the fecal samples of colorectal cancer by ITS sequencing (Gao et al., 2017b). Recently, a larger cohort study based on metagenomic sequencing found a significantly increased relative abundance of *Malasseziomycetes* and decreased proportion of *Saccharomycetes* in colorectal cancer (Coker et al., 2019). However, the gut mycobiota composition was affected by many factors, such as diet, antibiotics, and disease state. The demonstration of gut mycobiota alteration of different background patients and accurate taxonomic characterization was also needed for the comprehensive analysis of colorectal cancer.

Here, we presented a systemic analysis of gut mycobiota in colorectal cancer, adenoma, and healthy controls with a convincing sample size by metagenomic sequence. In addition, we compared the diversity index of gut mycobiota in the three groups and characterized the signatures of gut mycobiota in colorectal cancer at different taxonomic levels by metagenomic sequencing. Finally, we established a diagnosis panel by selecting important fungal biomarkers ranked by random forest, which might be helpful in the early detection of colorectal cancer in the clinical setting.

## Methods

### Enrollment of Patients

Colonic adenoma and colorectal cancer (CRC) patients included in the present study were enrolled at Shanghai Tenth People’s Hospital. The detailed characteristics of all the enrolled patients and healthy controls and the included and excluded criteria were described previously (Gao et al., 2021). Briefly, patients who had exposure to antibiotics, probiotics, and prebiotics within 1 month were excluded. Patients who had gastrointestinal surgery history or diagnosed with acute or chronic diarrhea and hepatitis were also excluded in the present study. All the individuals provided informed consent. The Ethics Committee of Shanghai Tenth People’s Hospital approved the study protocol. All the procedures performed during the study followed the Declaration of Helsinki and its later amendments.

### Sample Collection and Metagenomic Sequencing

Fecal samples were obtained from all subjects, transported to the lab, and stored immediately at −80°C. The details of DNA extraction and metagenomic sequencing were described previously (Gao et al., 2021). Briefly, genomic DNA was extracted, and libraries were established according to the Illumina manifestation instruction. Next, raw reads were preprocessed and filtered to exclude adaptor contaminated reads and low-quality reads. Finally, an average of 93.3% of high-quality reads was obtained as the clean reads for further analysis.

### Taxonomic Annotation and Statistical Analysis

The clean reads were assigned to microbial taxa by using the k-mer-associated algorithms as described before (Gao et al., 2021). Gene sequences were constructed by SOAPalign2.21. The relative abundances of genes were generated following Qin et al. (2014) Random forest (randomForest package in R) was applied to build the classifier based on the relative abundance of fungal species. SparCC was used to construct the associations among different fungal species. Linear discriminant analysis effect size (LEfSe) was used to identify the significantly dominated fungi in each group (Segata et al., 2011). Only two-sided *p*-values <0.05 were displayed in the network. The predictive model for early detection of colorectal cancer was estimated by 10-fold cross-validation. The non-parametric Wilcoxon test was used to analyze different taxonomic levels of fungi. The Benjamini–Hochberg method was used for adjustment for multiple comparisons. The adjusted *p*-value (false discovery rate, FDR) <0.05 was considered statistically significant.

### Data Access

The metagenomic sequence data sets have been deposited in the NCBI Sequence Read Archive (SRA) with accession numbers PRJNA706060 and PRJNA514108 (Gao et al., 2021).

## Results

### Clinical Characteristics of Enrolled Patients and Healthy Controls

A total of 71 CRC patients, 63 adenoma patients, and 91 healthy controls were finally included in the study, as described before (Gao et al., 2021). All these individuals in the three groups were comparable regarding sex, age, and BMI (*p* > 0.05) (**Table 1**). Most of the CRC patients were diagnosed with rectal cancer. CRC patients with stages II and III, categorized by the American Joint Committee on Cancer staging system, accounted for 57%. The KRAS gene mutation rate was 49.3% in all CRC patients.

**Table 1:** The characteristics of the studied subjects in the present study (mean ± standard deviation).

	Colorectal cancer	Adenoma	Healthy controls	*p*-value
No. of individuals	71	63	91	
Male (%)	42 (59.15)	34 (53.97)	38 (41.76)	0.07
Age (years)	61.85 ± 10.96	63.22 ± 7.29	60.23 ± 5.06	0.06
BMI	22.96 ± 3.85	23.81 ± 3.03	23.32 ± 1.97	0.11
A/T/D/S/R^a^	16/5/2/8/40	/	/	/
Stage I/II/III/IV	9/30/27/5	/	/	/
KRAS mutation (%)	35 (49.3%)	/	/	/
CEA (ng/ml)^b^	25.05 ± 14.52	/	/	/
AFP (ng/ml)	2.99 ± 1.22	/	/	/
CA153 (U/ml)	10.37 ± 4.66	/	/	/
CA-125 (U/ml)	16.98 ± 19.91	/	/	/
CA199 (U/ml)^b^	34.59 ± 15.38	/	/	/
CA724 (U/ml)^b^	6.68 ± 1.66	/	/	/
CA50 (IU/ml)^b^	16.30 ± 5.61	/	/	/

CEA, carcinoembryonic antigen; AFP, alpha-fetoprotein.

a One patient had two cancers in both ascending colon and rectum, which was counted in both ascending colon cancer and rectal cancer: A, ascending colon; T, transversal colon; D, descending colon; S, sigmoid colon; R, rectum.

b Mean ± standard error of mean (SEM).

### Alterations of Mycobiota Richness and Diversity in Adenoma and Colorectal Cancer

**Figure 1:**
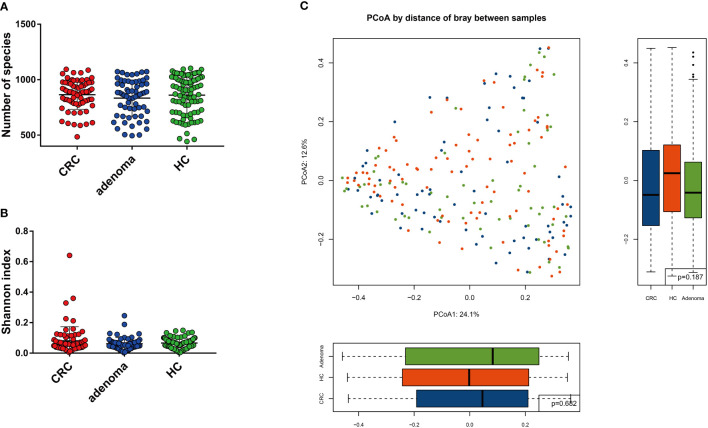
The mycobiota diversity comparison between colorectal cancer (CRC), adenoma, and healthy controls (HC). The number of species **(A)** and Shannon index **(B)** were similar between the three groups. Principal coordinate analysis of the mycobiota in the three groups **(C)**.

The number of species and α-diversity of gut mycobiota were compared in CRC, adenoma, and HC groups. No significant difference was observed among the three groups (*p* > 0.05) (**Figures 1A, B****)**. Principal coordinate analysis (PCoA) was also performed to evaluate β-diversity. PCoA1 and PCoA2 accounted for 24.1% and 12.6% of the variance, respectively (**Figure 1C**). Analysis of similarity did not show statistically significant differences among the three groups.

### Gut Mycobiota Dysbiosis in Adenoma and Colorectal Cancer

**Figure 2:**
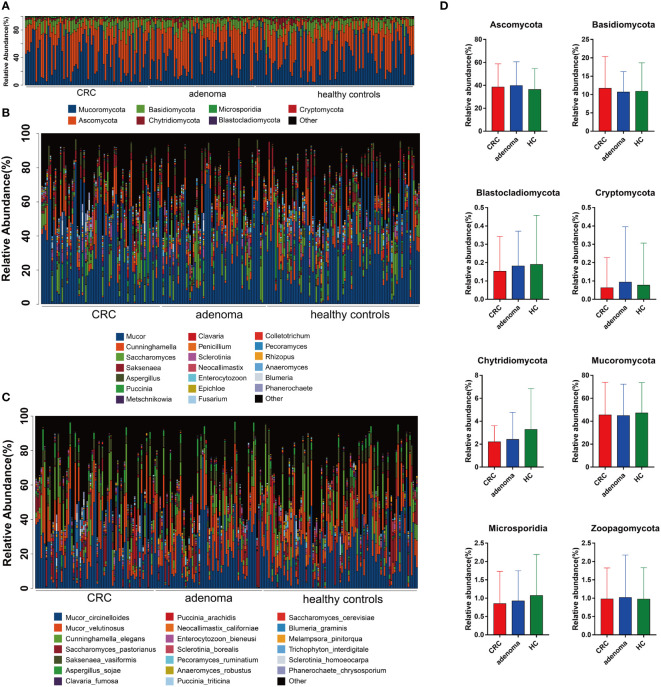
The taxonomy of gut mycobiota at the phylum **(A)**, genus **(B)**, and species **(C)** levels in colorectal cancer (CRC), adenoma, and healthy controls. The comparisons of eight mycobiota at the phylum level in CRC, adenoma, and healthy controls (HC) **(D)**. No significant changes were found between the three groups.

**Figure 3:**
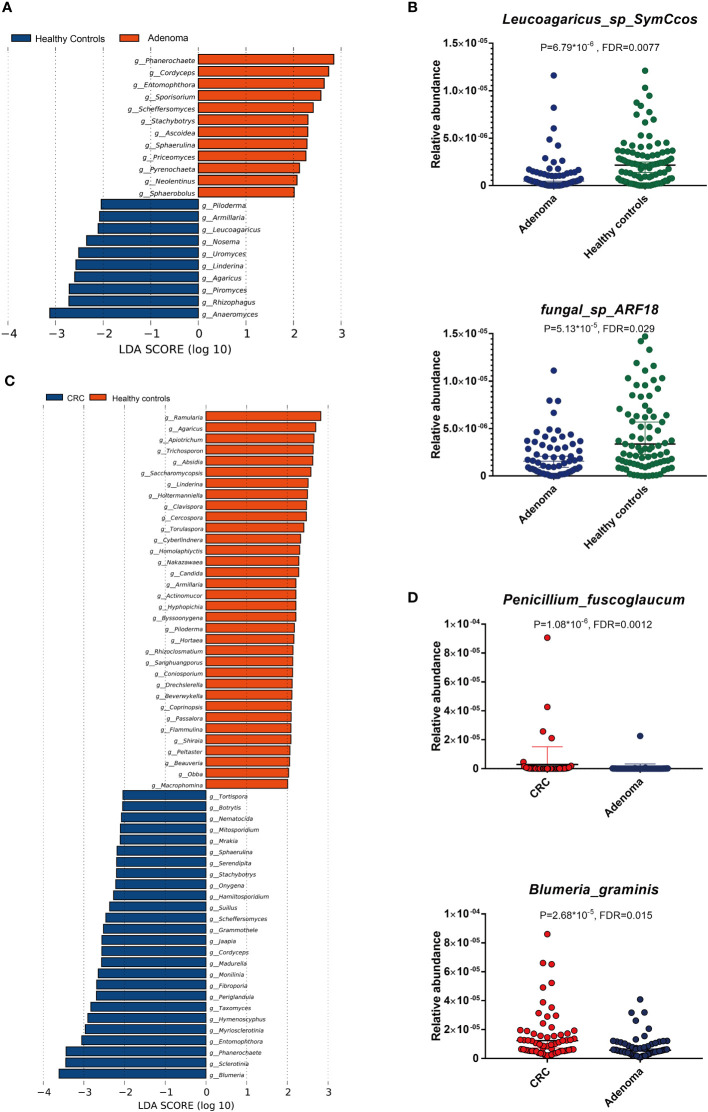
The linear discriminant analysis effect size (LEfSe) analysis revealed dominant gut mycobiota in adenoma and healthy controls **(A)**. The significant alterations of two mycobiota between adenoma and healthy controls by Wilcoxon rank-sum test **(B)**. The enriched gut mycobiota in colorectal cancer and healthy controls based on LEfSe analysis **(C)**. The significantly changed gut mycobiota between colorectal cancer and adenoma by Wilcoxon rank-sum test **(D)**. FDR, false discovery rate.

Compared with the healthy controls, the mycobiota taxonomic profiles were analyzed at the phylum, genus, and species levels in adenoma and colorectal cancer (**Figures 2A–C**). At the phylum level, eight main phyla were finally identified in all samples. Mucoromycota and Ascomycota were the major mycobiota at the phylum level in all samples. The relative abundances of Mucoromycota were 45.52% in CRC, 44.94% in adenoma, and 47.16% in healthy controls. The relative abundances of Ascomycota were 38.54% in CRC, 39.77% in adenoma, and 36.37% in healthy controls. No significant difference in the phyla was observed in the three groups (**Figure 2D**). The ratios of Ascomycota/Basidiomycota were also similar in the three groups (data not shown). At the genus level, *Anaeromyces* and *Phanerochaete* were the most significantly different genus enriched in adenoma and healthy controls by LEfSe, respectively (**Figure 3A**). *Malassezia_restricta* (*p* = 0.0014, FDR = 0.17) was enriched in the adenoma group at the species level, but no statistical difference was observed after statistical correction. Only two fungi, namely, *Leucoagaricus_sp_SymCcos* (*p* = 6.79 * 10^−6^, FDR = 0.0077) and *fungal_sp_ARF18* (*p* = 5.13 * 10^−5^, FDR = 0.029), showed significant decreased relative abundance in adenoma compared with healthy controls (**Figure 3B**). *Blumeria* dominated in CRC, while *Madurella* was enriched in adenoma by LEfSe (**Figure 3C**). *Penicillium_fuscoglaucum* (*p* = 1.08 * 10^−6^, FDR = 0.0012) and *Blumeria_graminis* (*p* = 2.68 * 10^−5^, FDR = 0.015) were found enriched in the CRC with a statistical difference when compared with adenoma by Wilcoxon test (**Figure 3D**).

**Figure 4:**
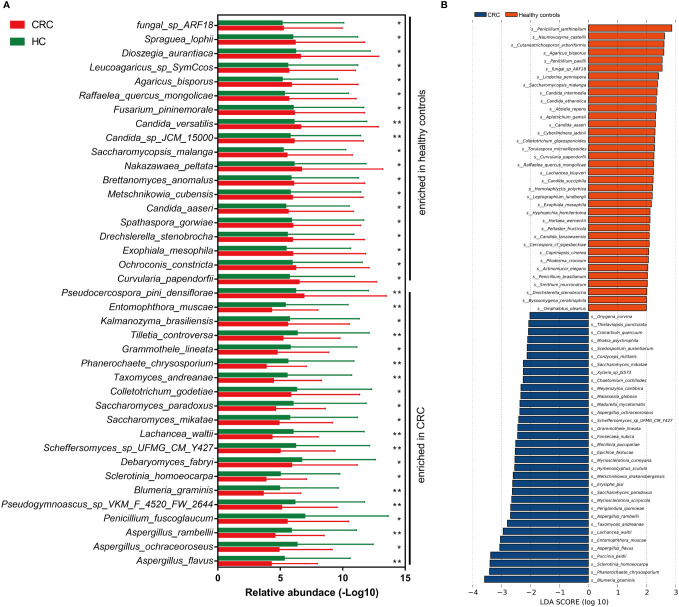
The alteration of gut mycobiota signatures in colorectal cancer (CRC). The mycobiota profiles that were enriched in CRC and healthy controls by Wilcoxon rank-sum test **(A)** and linear discriminant analysis effect size analysis **(B)**. *p < 0.05; **p < 0.01.

The altered gut mycobiota profiles of CRC were also evaluated, and a total of 39 fungi were found with statistical differences between CRC and healthy controls (**Figure 4A**). *Phanerochaete_chrysosporium*, *Lachancea_waltii*, and *Aspergillus_rambellii* were the top 3 fungi with the most significant differences that were enriched in CRC patients, while *Candida_versatilis*, *Pseudocercospora_pini_densiflorae*, and *Candida_sp_JCM_15000* were the top 3 fungi significantly decreased in CRC patients.

Besides, we also evaluated the CRC-dominated fungi by LEfSe. *Blumeria_graminis, Phanerochaete_chrysosporium, Sclerotinia_homoeocarpa, Puccinia_psidii, Aspergillus_flavus*, and *Entomophthora_muscae* were the main six enriched fungi in the CRC, while *Penicillium_janthinellum, Naumovozyma_castellii, Cutaneotrichosporon_arboriformis, Agaricus_bisporus, Penicillium_paxilli*, and *fungal_sp_ARF18* were the top 6 fungi in the healthy controls (**Figure 4B**). Interestingly, five of the top 6 dominated fungi at the threshold of LDA score of 3 by LEfSe were all identified as statistically different in CRC patients by Wilcoxon test.

### Diagnostic Model Based on Gut Mycobiota for Early Detection of Colorectal Cancer

**Figure 5:**
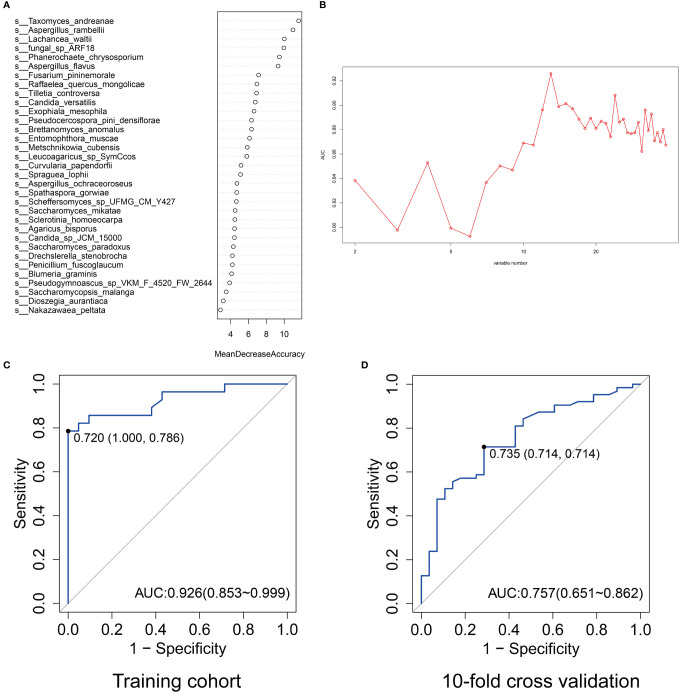
The ranking of importance of gut mycobiota by mean decreased accuracy at the species level **(A)**. The values of area under the curve (AUC) by different variable number of gut mycobiota **(B)**. The AUC ranked top when the corresponding variable number was 13. The AUC of the selected 13 gut mycobiota profiles for predicting colorectal cancer in the training cohort **(C)** and 10-fold cross-validation (**D**).

To identify the most important mycobiota in colorectal cancer, we used random forest to rank all the different mycobiota based on mean decreased accuracy. *Taxomyces_andreanae*, *Aspergillus_rambellii*, *Lachancea_waltii*, *fungal_sp_ARF18*, and *Phanerochaete_chrysosporium* were the top 5 mycobiota that ranked as most essential markers (**Figure 5A**). Based on the maximum value of area under the receiver-operating characteristics curve (AUROC), 13 mycobiota in importance ranking were selected as a novel diagnostic panel (**Figure 5B**). The AUROC of this panel in the testing model was 0.926 (95% confidence interval: 0.853–0.999), the sensitivity was 78.6%, and the specificity was 100% (**Figure 5C**). In the 10-fold cross-validation, the AUROC was 0.757 (95% confidence interval: 0.651–0.862), and both the sensitivity and specificity were 71.4% (**Figure 5D**).

### Mycobiota Correlations and Stage-Specific Distribution of Key Differed Markers in Colorectal Cancer

**Figure 6:**
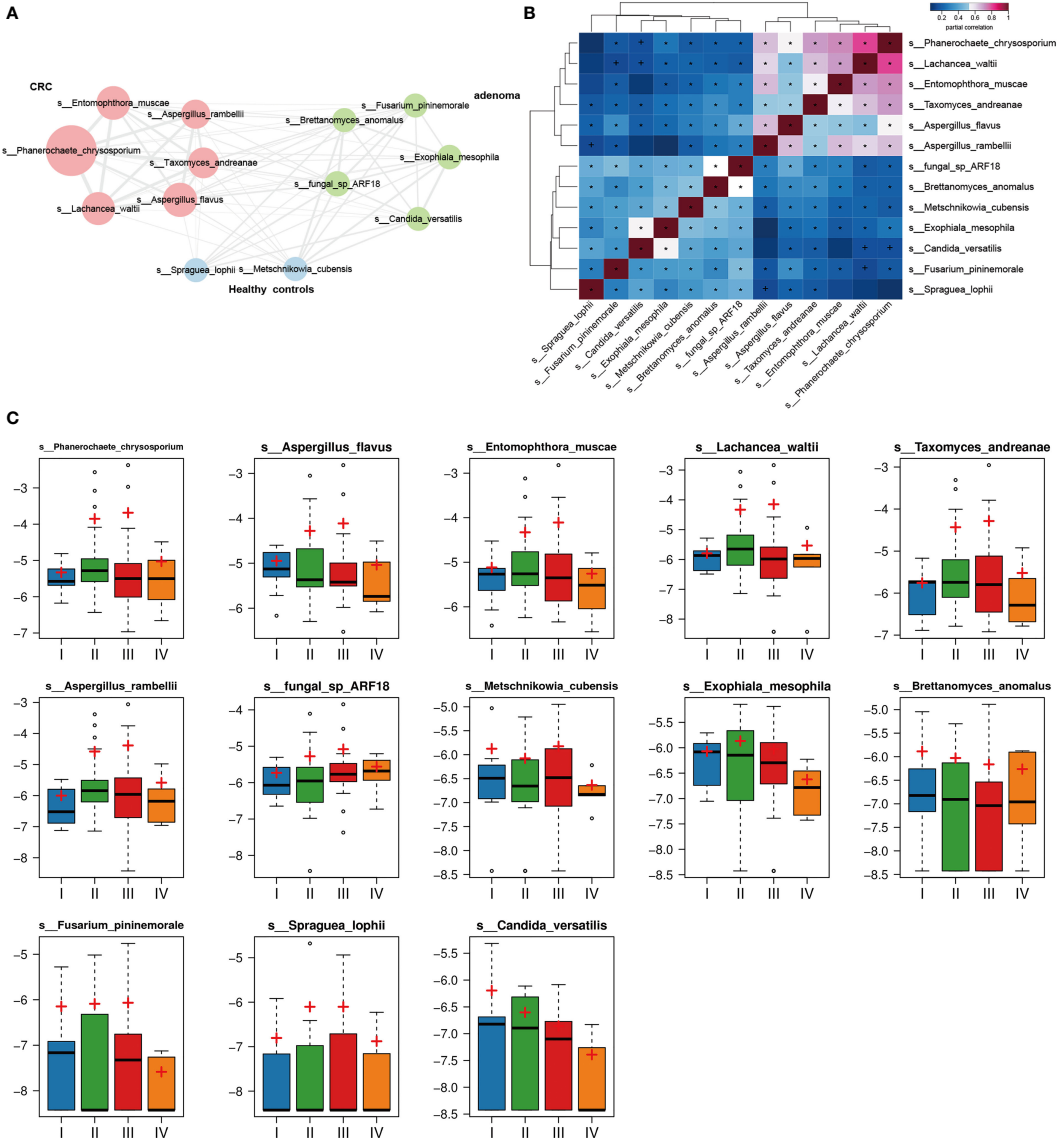
The 13 mycobiota network in colorectal cancer (CRC), adenoma, and healthy controls. All mycobiota showed positive correlations in the network **(A)**. The heatmap displayed positive relationships among the selected 13 gut mycobiota **(B)**. The relative abundances of the 13 mycobiota in different stages (stages I, II, III, and IV) of CRC **(C)**. *p < 0.05.

The mycobiota correlations of the key microbiota selected above were also evaluated in CRC, adenoma, and healthy controls. Interestingly, all 13 mycobiota showed positive associations (**Figures 6A, B****)**. The gut mycobiota in CRC had the most intense connections, followed by adenoma and healthy controls. *Lachancea_waltii* and *Phanerochaete_chrysosporium* showed the most significant association in CRC (*r* = 0.80, *p* = 1.55 * 10^−51^). *Phanerochaete_chrysosporium* was also positively associated with *Entomophthora_muscae* (*r* = 0.72, *p* = 6.00 * 10^−38^), *Taxomyces_andreanae* (*r* = 0.70, *p* = 2.91 * 10^−34^), and *Aspergillus_rambellii* (*r* = 0.64, *p* = 1.03 * 10^−27^) in CRC. *Aspergillus_rambellii* was positively associated with *Entomophthora_muscae* (*r* = 0.65, *p* = 1.87 * 10^−28^), *Aspergillus_flavus* (*r* = 0.65, *p* = 2.82 * 10^−28^), and *Lachancea_waltii* (*r* = 0.61, *p* = 1.25 * 10^−24^) in CRC. In adenoma, *fungal_sp_ARF18* was positively associated with *Brettanomyces_anomalus* (*r* = 0.53, *p* = 6.96 * 10^−18^). In the healthy controls, *Metschnikowia_cubensis* was positively associated with *Spraguea_lophii* (*r* = 0.35, *p* = 4.42 * 10^−8^).

We also evaluated the relative abundances of the key 13 mycobiota in different stages of CRC (**Figure 6C**). No statistical differences of the mycobiota were found between the four stages of CRC.

## Discussion

**Figure 7:**
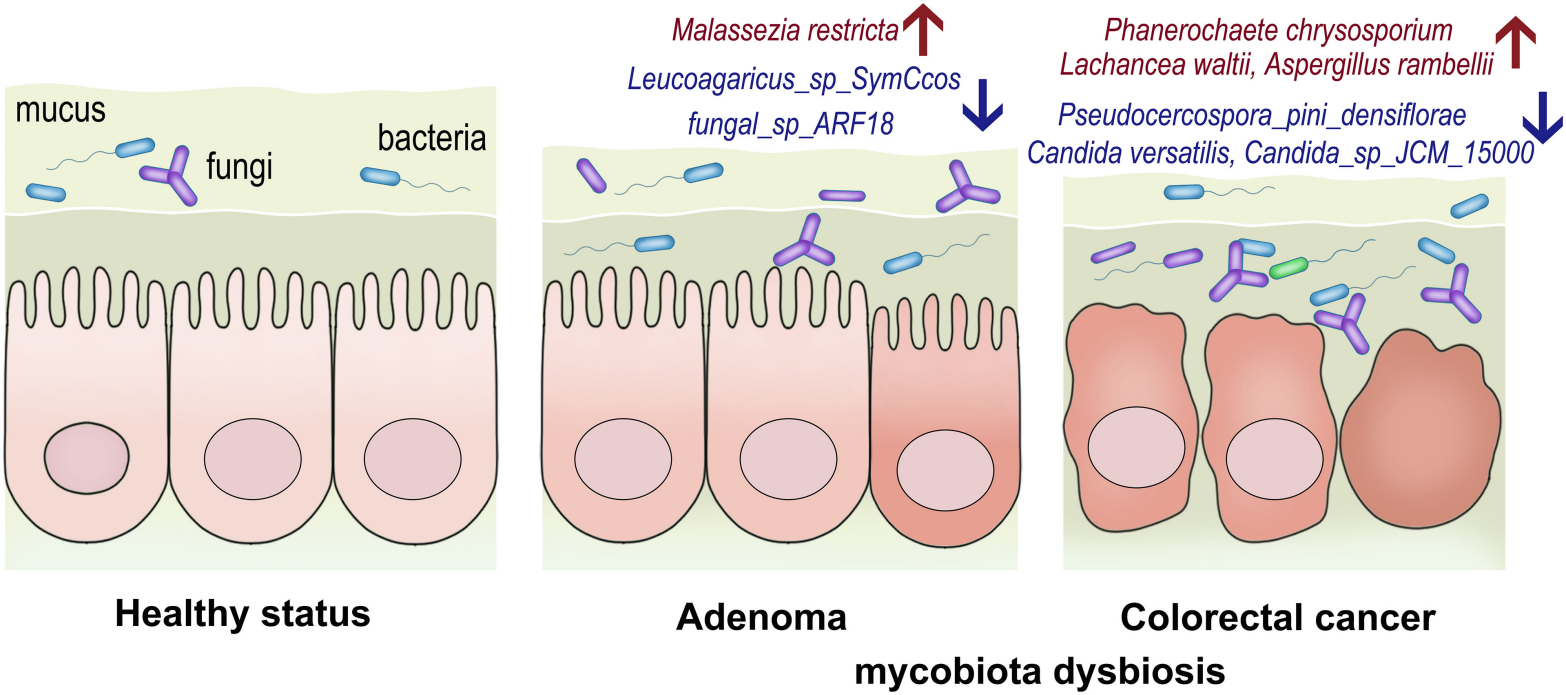
The conceptual figure to demonstrate the alterations of gut mycobiota in adenoma and colorectal cancer. The relative abundances of *Malassezia restricta* increased, while *Leucoagaricus_sp_SymCcos* and *fungal_sp_ARF18* significantly decreased in adenoma. *Phanerochaete_chrysosporium*, *Lachancea_waltii*, and *Aspergillus_rambellii* were the top 3 fungi that were significantly enriched in colorectal cancer, while *Candida_versatilis*, *Pseudocercospora_pini_densiflorae*, and *Candida_sp_JCM_15000* were dominant in the healthy controls.

The gut mycobiota plays a vital role in maintaining the healthy status of human beings. Our study presented the alteration of gut mycobiota and established the early diagnosis for CRC based on high-throughput sequencing, providing more sophisticated and convincing evidence on the potential roles of gut mycobiota in adenoma and CRC (**Figure 7**).

Nowadays, more and more studies have begun investigating the underestimated roles of gut mycobiota in colorectal cancer. Deciphering the mycobiota architecture was the first and essential step to uncover their potential roles in the occurrence and development of colorectal cancer. The altered signatures of gut mycobiota in colon polyp or adenoma and colorectal cancer were displayed by internal transcribed spacer sequencing (Luan et al., 2015; Gao et al., 2017b). Opportunistic fungi such as *Trichosporon* and *Malassezia* were found enriched in CRC. However, after applying metagenomics sequencing, more detailed information on fungi dysbiosis could be obtained. Coker et al. identified Malasseziomycetes dominant in CRC and fungal biomarker panel for CRC diagnosis, which showed an excellent discriminative capacity (Coker et al., 2019). The present study found several CRC-associated mycobiota. For instance, *Aspergillus*, including *Aspergillus rambellii*, *Aspergillus ochraceoroseus*, and *Aspergillus flavus*, were significantly enriched in CRC. This was consistent with a previously published study (Coker et al., 2019). *Aspergillus flavus* was reported to be involved in the production of *aflatoxin*, targeting aryl hydrocarbon receptor to mediate the expression of P450 to induce hepatocellular carcinoma process, indicating that these species seemed to have an underestimated relation with colorectal cancer (Sharma et al., 2021; Zhu et al., 2021a). *Debaryomyces fabryi* was also significantly enriched in CRC. A previous study demonstrated that *Debaryomyces* dominated in inflamed mucosal tissues of Crohn’s disease and impaired mucosal healing *via* myeloid-type I interferon–CCL5 axis (Jain et al., 2021). Intestinal mucosal inflammation, a detrimental consequence of antibiotics and *Debaryomyces* outgrowth, was also associated with mycobiota dysbiosis in the gut (Chiaro and Round, 2021). Whether *Debaryomyces* had a close implication with colonic carcinogenesis was still underestimated. We also noticed that *Saccharomyces* species, including *Saccharomyces paradoxus* and *Saccharomyces mikatae*, were also enriched in CRC. *Saccharomyces* consisted of at least eight species, and some species had been regarded as agents responsible for invasive infection (Enache-Angoulvant and Hennequin, 2005). However, rare studies reported the associations between *S. paradoxus* and *S. mikatae* in CRC. Of note, we also found that *fungal_sp_ARF18* was the only fungi that was enriched in both adenoma and colorectal cancer patients. The role of this fungi was still underestimated in human health and disease so far.

The potential reasons might be due to the limited culture technology and the lack of a high-throughput sequencing application in identifying candidate fungi. *Entomophthora muscae*, a fungal pathogen, was also enriched in CRC in the present study. This species was found to evade the nervous system and modulate the behavior of *Drosophila melanogaster*, but there was minimal evidence that demonstrated its role in human disease, especially in cancer (Elya et al., 2018). However, these findings also need to be confirmed in the future. Other unidentified fungi in the present study had been reported to be closely related to colon tumorigenesis. For example, *Candida albicans* contributed to colon cancer by glycolysis in macrophages, thus triggering the interleukin-22 secretion from innate lymphoid cells (Zhu et al., 2021b).

Nevertheless, the present study did not identify the significant enrichment of *C. albicans* in CRC or adenoma. In contrast, we found a considerable decrease of other *Candida* species, such as *Candida versatilis*, *Candida aaseri*, and *Candida_sp_JCM_15000*, in CRC. *Candida versatilis*, a yeast resistant to salt, was used for soy sauce fermentation (Ruan et al., 2019). Some salty food had been indicated to be a strong risk factor associated with colorectal cancer (Takachi et al., 2010). Therefore, we wonder if there are any potential relations between the unproven beneficial effect of *C. versatilis* and gut health. In addition, *Saccharomyces malanga*, a yeast that functioned in the fermentation of the traditional Chinese baijiu and rice wine, was also elevated in healthy controls compared with CRC (Wang et al., 2020).

In conclusion, our study elucidated the dysbiosis signatures of adenoma and colorectal cancer and demonstrated the diagnostic values of gut mycobiota for colorectal cancer. Moreover, we deciphered the CRC-associated gut mycobiota at the species level and their interactions in different statuses, which could contribute to understanding the roles in colonic carcinogenesis. However, of course, a tremendous amount of effects and extensive cooperation are needed to illustrate the gut mycobiota mystery in colorectal cancer, especially the mechanisms in the future.

## Data Availability Statement

The datasets presented in this study can be found in online repositories. The names of the repository/repositories and accession number(s) can be found below: https://www.ncbi.nlm.nih.gov/sra, accession ID: PRJNA706060 and PRJNA514108.

## Ethics Statement

The studies involving human participants were reviewed and approved by the Ethics Committee of Shanghai Tenth People’s Hospital. The patients/participants provided their written informed consent to participate in this study. Written informed consent was obtained from the individual(s) for the publication of any potentially identifiable images or data included in this article.

## Author Contributions

HQ, CC, and RY take responsibility for the integrity of the work as a whole, from inception to the publication of the article. RG wrote the manuscript. RG, KX, YZ, HZ, and MW performed the data check and analysis. YZ, LH, XW, JS, and LY helped collect all the subject information and samples. RG, KX, YZ, HZ, and RY performed the visualization and interpretation of data. HQ performed the sample sequencing and analysis. RG and HQ designed and guided the whole study. All authors contributed to the article and approved the submitted version.

## Funding

This work was supported by grants from the National Natural Science Foundation of China (No. 81972221), Training program of the National Natural Science Foundation of China of Shanghai Tenth People’s Hospital (SYGZRPY2017024), “Climbing” plan of Shanghai Tenth People’s Hospital (2018SYPDRC030), and Special Project of Clinical Research on Health Care Industry of Shanghai Municipal Health Commission (20194Y0483).

## Conflict of Interest

The authors declare that the research was conducted in the absence of any commercial or financial relationships that could be construed as a potential conflict of interest.

## Publisher’s Note

All claims expressed in this article are solely those of the authors and do not necessarily represent those of their affiliated organizations, or those of the publisher, the editors and the reviewers. Any product that may be evaluated in this article, or claim that may be made by its manufacturer, is not guaranteed or endorsed by the publisher.
